# Exploring the impact of a decision support intervention on vascular access decisions in chronic hemodialysis patients: study protocol

**DOI:** 10.1186/1471-2369-12-7

**Published:** 2011-02-03

**Authors:** Mary Ann Murray, Alison Thomas, Ron Wald, Rosa Marticorena, Sandra Donnelly, Lianne Jeffs

**Affiliations:** 1 The Ottawa Hospital, Riverside Campus, 1967 Riverside Drive, Ottawa ON, K1BH 7W9, Canada; 2 St Michael's Hospital, 30 Bond Street, Toronto, ON, M5B 1W8, Canada; 3 St Michael's Hospital, 61 Queen Street East, Suite 7-030, Toronto, ON, M5C 2T2, Canada

## Abstract

**Background:**

In patients with Stage 5 Chronic Kidney Disease who require renal replacement therapy a major decision concerns modality choice. However, many patients defer the decision about modality choice or they have an urgent or emergent need of RRT, which results in them starting hemodialysis with a Central Venous Catheter. Thereafter, efforts to help patients make more timely decisions about access choices utilizing education and resource allocation strategies met with limited success resulting in a high prevalent CVC use in Canada. Providing decision support tailored to meet patients' decision making needs may improve this situation. The Registered Nurses Association of Ontario has developed a clinical practice guideline to guide decision support for adults living with Chronic Kidney Disease *(Decision Support for Adults with Chronic Kidney Disease*.) The purpose of this study is to determine the impact of implementing selected recommendations this guideline on priority provincial targets for hemodialysis access in patients with Stage 5 CKD who currently use Central Venous Catheters for vascular access.

**Methods/Design:**

A non-experimental intervention study with repeated measures will be conducted at St. Michaels Hospital in Toronto, Canada. Decisional conflict about dialysis access choice will be measured using the validated SURE tool, an instrument used to identify decisional conflict. Thereafter a tailored decision support intervention will be implemented. Decisional conflict will be re-measured and compared with baseline scores. Patients and staff will be interviewed to gain an understanding of how useful this intervention was for them and whether it would be feasible to implement more widely. Quantitative data will be analyzed using descriptive and inferential statistics. Statistical significance of difference between means over time for aggregated SURE scores (pre/post) will be assessed using a paired t-test. Qualitative analysis with content coding and identification of themes will be conducted for the focus group and patient interview data.

**Discussion:**

Coupling the SURE tool with a decision support system structured so that a positive test result triggers providers to help patients through the decision-making process and/or refer patients to appropriate resources could benefit patients and ensure they have the opportunity to make informed HD access choices.

## Background

Chronic Kidney Disease (CKD) a serious long term illness affecting more than two million Canadians, is a major public health problem[1]. Patients with CKD stage 5 D - also referred to as end-stage kidney failure - require renal replacement therapy (RRT). Diabetes is the leading cause of Stage 5 CKD and 54% of patients requiring RRT in 2007 were over the age of 65. Between 1998 and 2007, there has been a 51% increase in the number of patients with CKD stage 5 D receiving hemodialysis (HD)[2]. The estimated annual cost of hospital based HD (inpatient or outpatient clinic) in Canada per patient in 2002 was $59,476.00 [3].

Adults living with Stage 5 CKD face numerous decision points [4]. These patients often experience uncertainty when considering the best course of action among different options for treatment and care. This uncertainty is called decisional conflict [5]. The RNAO nursing BPG *Decision Support for Adults Living with Chronic Kidney Disease *[6] outlines the importance of screening for decisional conflict and applying decision support strategies to address unmet decision making needs in this high risk population.

A major decision for patients, with Stage 5 CKD receiving or who are planning to initiate HD therapy is about dialysis vascular access. Evidence-based standards of care recommend an arteriovenous fistula (AVF) as the optimal access due to its longevity and lower complication rates compared to central venous catheters (CVC)[7]. Many CKD patients are followed by an interprofessional team, and education related to RRT modality options and vascular access planning are key components of the care provided. Despite this, patients often defer modality choice during pre-dialysis stages, or may not receive pre-dialysis care, leading to acute HD initiation with an obligatory CVC as the vascular access in an urgent or emergent situation. This contributes to a CVC rate in Canada that is well above international standards for best CKD care with a corresponding increased risk of morbidity and mortality for patients [8]. Current estimates suggest that CVCs are used for initial HD access in 70% of cases[9]. Once a CVC has been inserted as a vascular access, about 33% of Canadian patients continue to use a CVC as their permanent vascular access putting them at increased risk for morbidity and mortality[9].

Reported reasons for the high incidence and prevalence of CVCs in Canada include late referral to nephrologists and fewer vascular surgeons per capita (who arrange for and perform AVF surgery) as compared with the USA and Europe [9]. Efforts to optimize the timeliness of patient decisions about access through patient and staff education and resource allocation strategies have been unsuccessful in addressing the high CVC rates in Canadian HD units [8]. Reports from systematic reviews confirm that concerns about impact of decisions on others and on quality of life; wish to maintain normalcy; influence of others; and personal assessments of perceived risk/benefits affect patients' decisions about CKD treatment options [4,10]. Added to this is the difficulty in translating population based probabilities of risks and benefits to the individual level which also contributes to uncertainty when making health decisions. Consequently, patients can experience decisional conflict due to knowledge gaps, lack of clarity about what matters most to avoid or achieve among the possible outcomes of options under consideration or have support needs which in turn affects their readiness to participate in decision making. A process that helps patients explicitly consider their choices, information needs, values and preferences when making decisions about HD access could help to address these unmet decision making needs.

Decision support interventions help patients a) prepare for decision making and b) to arrive at a quality decision informed by both evidence and patient values [11]. In practice areas other than CKD, decision support interventions have been found to be effective in reducing decisional conflict. The RNAO BPG for *Decision Support for Adults with CKD *[6]suggests several evidence based approaches to help mediate patients' decisional conflict. However, to our knowledge these recommendations have not been tested in clinical practice. As well, the impact of a targeted decision support intervention on patient and health service outcomes is unknown.

## Purpose & Objectives

The purpose of this study is to determine the impact of implementing selected recommendations from the *RNAO Decision Support for Adults with Chronic Kidney Disease *(CKD) best practice guideline (BPG) [1]on priority provincial targets for hemodialysis (HD) access sites in patients with Stage 5 CKD requiring dialysis (Stage 5D) who currently use central venous catheters (CVC) as their HD access. Specific research objectives will be to:

1) Identify the prevalence of decisional conflict in a cohort of patients with CKD Stage 5 D who receive HD via CVC access;

2) Identify the most frequently reported sources of decisional conflict identified by patients with CKD Stage 5 D who receive HD via CVC access;

3) Determine the impact of tailored decision support interventions identified from decisional conflict screening on HD access decisions among a cohort of patients with CKD Stage 5 D who receive HD via CVC access; and

4) Identify the acceptability, feasibility of such an approach from the perspective of patients with CKD Stage 5 D who receive HD via CVC access and providers.

### Guiding Theoretical Framework

The Ottawa Decision Support Framework (ODSF), which underpins the Decision Support Guideline for CKD[12], guides study measures and outcomes. The ODSF proposes that decisional needs [uncertainty, knowledge, values clarity, support, personal characteristics] strongly influence the quality of decisions patients make and that providers can improve the quality of those decisions by providing decision support to address decisional needs [clarify decisional needs, provide facts, probabilities, clarify values, support/guide deliberation, monitor/facilitate progress].

## Methods/Design

### Design

A prospective quasi-experimental intervention study with repeated measures (baseline and post decision support intervention) over an 18 month period will be conducted). This study incorporates quantitative and qualitative approaches to triangulate findings and provide a fuller perspective[13]. The use of qualitative and quantitative data will contribute to our understanding of decisional conflict and potential modifiers in the context of HD access decisions for patients with Stage 5 CKD [14].

### Intervention

Decisional conflict can be screened with the 4 item SURE tool. Based on core concepts of the validated Ottawa Decisional Conflict Scale[15] the SURE tool has been found suitable for screening decisional conflict in French and English-speaking patients with a variety of health conditions[16]. Four questions target sources of decisional conflict (feeling uncertain, feeling informed, feeling clear about values and feeling supported in decision making). Responses are scored as yes (score = 1) or no (score = 0). Scores of less than 3 indicate decisional conflict. Coupling the SURE tool with a decision support system structured so that a positive test result (scores of 3 or less) triggers providers to help patients through the decision-making process through a collaborative effort involving the interprofessional health care team and/or refer patients to appropriate resources could benefit patients and ensure patients have the opportunity to make informed HD access choices.

### Setting

The study will take place at Toronto's St. Michael's Hospital. St Michael's is a tertiary care teaching and research hospital with more than 5,000 staff, 600 physicians and 1,100 students, which is affiliated with the University of Toronto. With more than 450 inpatient beds and extensive outpatient clinics, the Hospital has a large kidney disease program including an in-centre hemodialysis unit providing outpatient and inpatient hemodialysis unit where 235 patients with Stage 5 D CKD receive care.

### Participants

A purposive sampling strategy will be used to ensure that the study sample will be representative of the level of care within the renal patient population. Participants will be recruited from the following categories: a) patients (n = 40) receiving HD; b) professionals from the interprofessional team (n = 10-12) providing direct patient care within and/or directly associated with HD delivery; and c) managers and educators (n = 4-6) with varying levels of influence in the renal program practice environment (e.g., managers, educators, senior leaders influencing practice). Eligible patient participants include Stage 5 CKD HD patients with CVCs who would be candidates for AVF who are receiving maintenance HD in the hemodialysis unit and who are able to communicate in English and who are judged mentally and physically able to participate by the HD care team. Eligible health professional participants include nurses, physicians, dieticians, pharmacists, and social workers. Eligible managers and educators include nurse practitioners, nurse educators, vascular access coordinators, clinical leader managers, and the program director.

### Data Collection and Procedures

Sessions outlining study information and procedures will be held in staff meetings and rounds. Education based on the RNAO BPG for Decision Support in CKD[1] recommendations will be provided to HD clinic nurses by the HD Nurse Practitioner (a member of the CKD BPG development team). Table 2 summarizes the relationship between research objectives, data collection and analysis. In line with our research objectives, study procedures include three main phases:

**Phase 1: Identify the prevalence of decisional conflict ***(research objective 1&2: BPG recommendation 1,2, 3,4,5)*. Nurses in the HD clinic will provide information about the study to eligible patients. The research coordinator will obtain informed consent from patients indicating an interest in participating. The HD nurse will then screen for decisional conflict using the SURE tool. Results of the screening (presence or absence of decisional conflict and source(s)) will be verbally communicated to the interprofessional team and will be recorded in the patient health record. After the source(s) of decisional conflict has been pinpointed, a decision support intervention will be developed using the Decision Support for Adults Living with Chronic Kidney Disease BPG. This intervention will be tailored to meet the identified need and will be led by an Advanced Practice Nurse who specializes in CKD Stage 5 D care. For instance, the nurse will help the patients through a decision-making process (i.e.: provide facts; discuss with patient what is personally important for them to achieve or avoid; clarify what resources patient needs to make a decision) and/or refer patients to appropriate resources or members of the interprofessional team (i.e.: facilitate team conference/family meeting; refer to social worker). The SURE tool will be repeated following the decision support intervention to evaluate the intervention and plan next steps. Intervention details, outcomes and planned next steps will be documented in the patient health record.

**Phase 2: Determine the impact of tailored decision support interventions ***(research objective 3: BPG recommendation 4,7,8)*. We will aggregate and compare the pre and postintervention SURE scores. Using chart review and our Program's HD Vascular Access Database, congruence between identified preferred choice following the intervention and actual HD access will be assessed. Interviews based on previous research related to patient decision making needs will be conducted with patient participants [17].

**Phase 3: Identify the acceptability, feasibility of such an approach **(research objective 4: BPG recommendation 7,8). We will conduct focus groups with members of the interprofessional team who have been directly or indirectly involved with patient participants. A focus group will also be held with the renal program management and educator group. Focus group guides will be adapted from our previous research about barriers, facilitators and implementation strategies for decision support interventions [18].

### Data Analysis

Quantitative data will be analyzed using descriptive and inferential statistics. Statistical significance of difference between means over time for aggregated SURE scores will be assessed using a paired t-test. The estimated sample size for the t-test is based on a test for differences in mean scores of decision conflict (SURE scores) pre and post intervention. An effect size of .70 requires n = 32/group, when alpha error = 0.05 and beta error = 0.20[19]. To accommodate attrition we plan to recruit 40 patients. Congruence between identified preferred HD access choice following intervention and actual HD access will be analyzed using Pearson r correlation. Qualitative analysis with content coding and identification of themes will be conducted for the focus group and patient interview data using well established criteria to maintain trustworthiness and credibility of analysis processes and findings [20].

### Ethical Considerations

Ethics approval has been obtained from St Michaels Research Ethics Board. All participants will receive study information letters and informed consent forms.

### Dissemination

Patients and providers will be directly involved in providing feedback about the feasibility and acceptability of the SURE tool and decision support intervention on the process of care thereby making the findings from this study more relevant to the organization's processes of care. Intended users of our research results include kidney health teams, educators, organizational managers and senior leaders and health policy makers interested in addressing barriers to HD access in clinical practice. As well, members of the research team have extensive and relevant experience and connections with the intended users. End of grant activities will include dissemination of study results: a) at conferences (scientific and professional) with themes related to shared decision making, interprofessionalism and renal policy; and b) on the website of institutions where team members are located. The research protocol and study results will be published in peer reviewed open-accessed journals.

## Discussion

Patient uncertainty related to renal treatments and particularly for vascular access is well known [21,22]. This will be the first study to evaluate the impact of a patient decision support intervention designed to help providers identify sources of decisional conflict patients are experiencing. Tailored decision support tailored to address decision making needs can then be provided. This reproducible, portable intervention addresses a key policy mandate regarding patient involvement in care planning set out by health providers such as the Ontario Ministry of Health in Canada.

This is a pragmatic study with relatively inclusive entry criteria. There are three identified benefits of the proposed research study: 1) improved clinical practice through standardized assessment for decisional conflict for adults living with chronic kidney disease; 2) provision of a practical approach to engage patients in decisions about vascular access and enhance the quality of their decision making and; 3) improved communication among health care team members. Moreover, the SURE assessment tool and other tools tested will provide health care practitioners with common inter-professional measures to identify decisional conflict with those living with chronic kidney disease. The SURE assessment tool will facilitate the communication of the patient's decisional conflict among the health care team members. With a standardized, repeatable measure of decisional conflict, the patient and the health care team members will be able to determine appropriate interventions, the effectiveness of those interventions and enhance communication among team members in regards to decisional conflict and appropriate care modalities. Participation of clinicians in the research process will foster a sense of inquiry and engagement and will ensure clinical relevance of the measure's content, which will facilitate its future use in the practice setting. Should this approach show promise this initial step will provide the foundation for large scale future testing and generalizability of the assessment tools and interventions. The success of this study and future studies will lead to improved patient care. Further, this study will provide corroboration of current RNAO best practice guidelines and lead to future recommendations for guideline implementation.

## Competing interests

The authors declare that they have no competing interests.

## Authors' contributions

MAM and AT conceived of the study and drafted the manuscript. LJ participated in its design and coordination and helped to draft the manuscript. SD, RW and RM are providing support during the implementation of the study and analysis of study data and have contributed to manuscript preparation. All authors read and approved the final manuscript.

## Pre-publication history

The pre-publication history for this paper can be accessed here:

http://www.biomedcentral.com/1471-2369/12/7/prepub
